# Recessive Mutations in *TRMT10C* Cause Defects in Mitochondrial RNA Processing and Multiple Respiratory Chain Deficiencies

**DOI:** 10.1016/j.ajhg.2016.03.010

**Published:** 2016-04-28

**Authors:** Metodi D. Metodiev, Kyle Thompson, Charlotte L. Alston, Andrew A.M. Morris, Langping He, Zarah Assouline, Marlène Rio, Nadia Bahi-Buisson, Angela Pyle, Helen Griffin, Stefan Siira, Aleksandra Filipovska, Arnold Munnich, Patrick F. Chinnery, Robert McFarland, Agnès Rötig, Robert W. Taylor

**Affiliations:** 1 INSERM U1163, Université Paris Descartes-Sorbonne Paris Cité, Institut Imagine, 75015 Paris, France; 2 Institute of Neuroscience, Wellcome Trust Centre for Mitochondrial Research, Newcastle University, Newcastle upon Tyne NE2 4HH, UK; 3 Institute of Human Development, University of Manchester, Manchester M13 9WL, UK; 4 Central Manchester University Hospitals NHS Foundation Trust, Manchester Academic Health Science Centre, Manchester M13 9WL, UK; 5 Departments of Pediatric, Neurology and Genetics, Hôpital Necker-Enfants-Malades, 75015 Paris, France; 6 Institute of Genetic Medicine, Wellcome Trust Centre for Mitochondrial Research, Newcastle University, Newcastle upon Tyne NE1 3BZ, UK; 7 Harry Perkins Institute of Medical Research, Centre for Medical Research and School of Chemistry and Biochemistry, The University of Western Australia, Nedlands, WA 6009, Australia; 8 Medical Research Council Mitochondrial Biology Unit, Cambridge CB2 0XY, UK; 9 Department of Clinical Neurosciences, School of Clinical Medicine, University of Cambridge, Cambridge CB2 0SP, UK

## Abstract

Mitochondrial disorders are clinically and genetically diverse, with mutations in mitochondrial or nuclear genes able to cause defects in mitochondrial gene expression. Recently, mutations in several genes encoding factors involved in mt-tRNA processing have been identified to cause mitochondrial disease. Using whole-exome sequencing, we identified mutations in *TRMT10C* (encoding the mitochondrial RNase P protein 1 [MRPP1]) in two unrelated individuals who presented at birth with lactic acidosis, hypotonia, feeding difficulties, and deafness. Both individuals died at 5 months after respiratory failure. MRPP1, along with MRPP2 and MRPP3, form the mitochondrial ribonuclease P (mt-RNase P) complex that cleaves the 5′ ends of mt-tRNAs from polycistronic precursor transcripts. Additionally, a stable complex of MRPP1 and MRPP2 has m^1^R9 methyltransferase activity, which methylates mt-tRNAs at position 9 and is vital for folding mt-tRNAs into their correct tertiary structures. Analyses of fibroblasts from affected individuals harboring *TRMT10C* missense variants revealed decreased protein levels of MRPP1 and an increase in mt-RNA precursors indicative of impaired mt-RNA processing and defective mitochondrial protein synthesis. The pathogenicity of the detected variants—compound heterozygous c.542G>T (p.Arg181Leu) and c.814A>G (p.Thr272Ala) changes in subject 1 and a homozygous c.542G>T (p.Arg181Leu) variant in subject 2—was validated by the functional rescue of mt-RNA processing and mitochondrial protein synthesis defects after lentiviral transduction of wild-type *TRMT10C*. Our study suggests that these variants affect MRPP1 protein stability and mt-tRNA processing without affecting m^1^R9 methyltransferase activity, identifying mutations in *TRMT10C* as a cause of mitochondrial disease and highlighting the importance of RNA processing for correct mitochondrial function.

## Main Text

Mitochondrial respiratory chain deficiencies lead to insufficient ATP production from oxidative phosphorylation (OXPHOS), resulting in a wide range of clinical presentations broadly recognized as “mitochondrial disorders.” Mitochondrial diseases are genetically diverse, owing to the necessary expression, co-ordination, and activity of factors encoded by both the mitochondrial and nuclear genomes for proper mitochondrial function. The 16.6 kb human mitochondrial DNA (mtDNA) encodes only 22 tRNAs, 2 rRNAs, and 13 polypeptides that are essential components of four of the five OXPHOS complexes.^1^ The remaining subunits of the respiratory complexes and all of the factors involved in mtDNA expression and maintenance are encoded by the nuclear genome, synthesized in the cytosol, and imported into mitochondria. Thus, there are a large number of potential genetic causes of mitochondrial disease, which has often complicated attempts to identify the correct genetic diagnosis. The advent of next generation sequencing has greatly expanded the list of known gene mutations associated with mitochondrial disease,^2^ including several genes involved in mitochondrial (mt)-tRNA processing and maturation.^3^, ^4^

In mammalian mitochondria, all mt-tRNAs required for mitochondrial protein synthesis are encoded by the mitochondrial genome. Transcription of mtDNA produces long polycistronic transcripts that require further processing. Most mitochondrial open reading frames are separated by at least one mt-tRNA gene, with the structure of mt-tRNAs acting as “punctuation” marks in the transcript^5^ prior to mt-tRNAs being excised at the 5′ end by the RNase P complex and at the 3′ end by the RNase Z enzyme. The mitochondrial RNase P in animals is composed of three proteins, MRPP1, MRPP2, and MRPP3^6^ (encoded by *TRMT10C* [MIM: 615423], *HSD17B10* [MIM: 300256], and *KIAA0391* [MIM: 609947], respectively), whereas RNase Z is encoded by a single gene, *ELAC2* (MIM: 605367).^7^, ^8^, ^9^, ^10^ In addition to cleavage from the polycistronic transcripts, mt-tRNAs undergo many further modifications, with at least 30 different modified residues reported.^3^, ^11^ One crucial modification is m^1^R9 methylation, which is probably important for the correct folding of most mt-tRNAs. In the case of mt-tRNA^Lys^, the unmodified in vitro transcript folds into an extended bulged hairpin,^12^ but with the sole modification of N1 methylation of adenosine 9 (m^1^A9), the tRNA adopts the classic cloverleaf structure.^13^ It has been demonstrated that MRPP1 and MRPP2 can form a stable sub-complex that is active as a methyltransferase and is uniquely able to methylate both adenosine and guanine nucleotides at position 9.^7^, ^14^ 19 of the 22 mt-tRNAs contain either A or G at position 9 and it is likely that all of these are subject to m^1^R9 methylation.^7^, ^11^, ^14^

We studied two children with suspected mitochondrial disease from unrelated families. Subject 1 (male) was the second child of healthy, non-consanguineous, white British parents, with a healthy older sister. He was born at term by a normal vaginal delivery after a normal pregnancy with a birth weight of 3.7 kg. He did not require resuscitation but was noted to be hypotonic and weak soon after birth. He fed poorly and gained weight slowly, partly due to gastro-esophageal reflux. Neonatal screening revealed significant hearing impairment subsequently confirmed to be sensorineural deafness. He was also found to have a raised plasma alanine transaminase of 439 U/L (normal range 4–45 U/L). Blood lactate levels ranged from 5 to 10 mmol/L (normal range 0.7–2.1 mmol/L) and his CSF lactate level was also elevated at 4.8 mmol/L. Ophthalmological examination, echocardiography, and an MRI were all normal, though the latter was of poor quality. There was a clinical suspicion of craniosynostosis and a lateral skull X-ray appeared to show fused sutures but no further investigations were undertaken. Ultrasound of the kidneys was normal. Blood spot acylcarnitine analysis and plasma biotinidase were normal, plasma amino acid analysis was normal with the exception of a raised alanine concentration, and urine organic acid analysis was normal apart from a raised lactate concentration. He deteriorated rapidly, requiring tube feeding, and at the age of 4 months suffered rhinovirus bronchiolitis requiring ventilatory support with CPAP. It proved impossible to wean him off ventilatory support and he died at 5.5 months of age after withdrawal of this support.

Subject 2 (female) was the second child of unrelated parents of Kurdish origin with a healthy older brother. She was born at term by caesarean delivery, after a normal pregnancy, weighing 3.05 kg. Hypotonia, poor sucking, and feeding difficulties were evident from early in the neonatal period and hyperlactatemia (7.4 mmol/L; normal range 0.90–1.70 mmol/L) with a high lactate to pyruvate ratio (170; normal range 30–50) was recorded at 1 month. She gained weight poorly and nasogastric feeding was commenced at 3 months. At 3.5 months, echocardiography demonstrated left ventricular hypertrophy and lumbar puncture revealed elevated CSF lactate (3.1 mmol/L; control < 2.2 mmol/L). She also had significantly impaired liver function (AST: 84 UI/L, normal range 15–60; ALT: 52 UI/L, normal range 7–40; gGT: 262 UI/L, normal range 6–25 UI/L). Brain MRI was undertaken at 2 months of age and was of poor quality but showed findings suggestive of bifrontal polymicrogyria. Acoustic oto-emissions were abnormal at 4 months, suggesting deafness. Unfortunately, auditory evoked potential was not done. She died at 5 months of age from respiratory distress.

**Figure 1:**
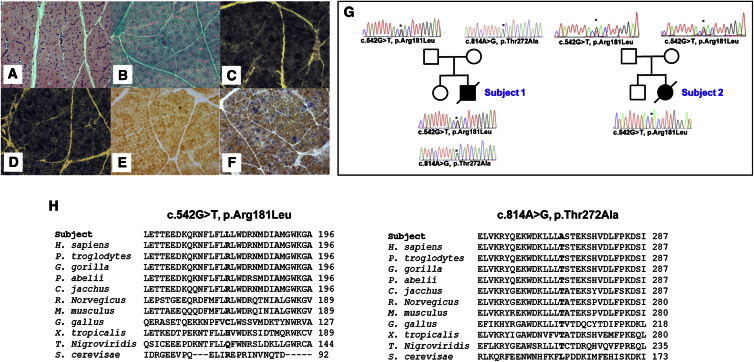
Autosomal-Recessive *TRMT10C* Variants Affect Evolutionarily Conserved Amino Acids and Are Associated with Mitochondrial Dysfunction (A–G) Histopathological analysis of skeletal muscle sections from subject 1 showing (A) hematoxylin and eosin (H&E) staining, (B) modified Gomori trichrome staining, (C) succinate dehydrogenase (SDH) histochemistry, (D) NADH-tetrazolium reductase histochemistry, (E) cytochrome *c* oxidase (COX) histochemistry, and (F) sequential COX-SDH histochemistry, which clearly illustrates a mosaic pattern of COX deficiency (G). Pedigree and sequencing chromatograms for the families of subject 1 (left) and 2 (right) showing segregation of biallelic *TRMT10C* variants. (H) Sequence alignment depicting the evolutionary conservation of the affected amino acids (bold).

Informed consent for diagnostic and research studies was obtained for both subjects in accordance with the Declaration of Helsinki protocols and approved by local Institutional Review Boards in Newcastle upon Tyne, UK, and Paris, France. Biochemical analysis of skeletal muscle samples identified clear mitochondrial enzyme defects involving both complex I and IV in both subjects, whereas complex III activity was normal in subject 1 but decreased in subject 2; both cases showed sparing of complex II activity (Table 1). Histopathological analysis of muscle from subject 1 revealed evidence of subsarcolemmal mitochondrial accumulation (ragged red fibers) and a mosaic pattern of cytochrome *c* oxidase (COX) deficiency (Figures 1A–1F).

Analysis of muscle DNA from both subjects excluded mtDNA abnormalities (mtDNA rearrangements and point mutations) and mtDNA copy number was shown to be normal in each case (data not shown). Whole-exome sequencing via previously described methodologies and bioinformatic filtering pipelines^2^, ^15^ identified biallelic variants in *TRMT10C* (MIM: 615423; GenBank: NM_017819.3; also known as *MRPP1* and *RG9MTD1*). Compound heterozygous c.542G>T (p.Arg181Leu) (ClinVar: SCV000264779.0) and c.814A>G (p.Thr272Ala) (ClinVar: SCV000264780.0) variants were identified in subject 1, whereas subject 2 was homozygous for the c.542G>T (p.Arg181Leu) variant, also identified in subject 1. Sanger sequencing was undertaken to validate the variants and confirm that these segregated with disease in each family (Figure 1G). Both identified *TRMT10C* variants are predicted to result in amino acid substitutions affecting evolutionarily conserved residues (Figure 1H) and are rare: the c.542G>T (p.Arg181Leu) variant is present on the ExAC database (10/120,324 alleles) and ESP6500 (1/11,824 alleles) whereas the c.814A>G (p.Thr272Ala) *TRMT10C* variant is absent on ExAC, ESP6500, and COSMIC. In silico predictions via SIFT, PolyPhen-2, and aGVGD suggest that the biophysico impact of the p.Arg181Leu substitution are relatively benign but that the proximity of the Arg181 residue to the TRM10-type domain (predicted from Met191) could hint at a crucial structural role that only an arginine residue can perform. In silico modeling of the *TRMT10C* variants via RaptorX and Phyre2 produced disparate predictions of MRPP1 protein structure and thus could not be used to indicate any potential misfolding as a consequence of the variants.

**Figure 2:**
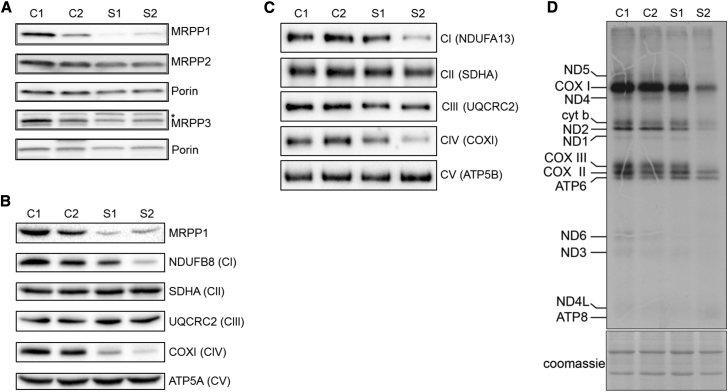
Mutant MRPP1 Protein Is Unstable and Leads to Impaired Mitochondrial Protein Synthesis Causing Multiple Respiratory Chain Defects (A) Western blot analyses of mitochondrial extracts from control and affected individuals using commercial MRPP1-, MRPP2-, and MRPP3-specific antisera. Porin (VDAC1) was used as a loading control. Asterisk (^∗^) indicates cross-reacting band. (B) Western blot analyses of protein extracts from affected fibroblasts showing decreased levels of MRPP1 and subunits of mitochondrial respiratory complexes I (NDUFB8) and IV (COXI and COXII). (C) Blue-native PAGE analysis of OXPHOS complex assembly using mitochondrial extracts in 1% DDM from control and subject fibroblasts (as described previously^16^) demonstrated decreased assembly of OXPHOS complexes I and IV and to a lesser extent complex III in subject fibroblasts. (A–C) Samples (25 μg protein) were fractionated through 4%–16% native gels (Thermo Fisher Scientific) transferred onto PVDF membranes and subjected to western immunoblotting. Subunits from individual OXPHOS complexes were detected using specific antibodies: complex I (NDUFA13), complex II (SDHA), complex III (UQCRC2), complex IV (COXI), and complex V (ATP5B). (D) In vitro labeling of de novo synthesized mitochondrial translation products with EasyTag EXPRESS^35^S Protein Labeling Mix (Perkin Elmer) followed by fractionation through a 17% SDS-Polyacrylamide gel and autoradiography (as described previously^32^). Coomassie brilliant blue staining of the gels was used to demonstrate equal loading.

To investigate the functional effects of the identified *TRMT10C* variants, Western blot and mitochondrial protein synthesis assays were performed in fibroblast cell lines derived from both affected individuals and age-matched control subjects. These data showed that the steady-state levels of MRPP1 were markedly decreased in the subject cell lines, suggesting that the variants affect the stability of the protein (Figure 2A). On the other hand, levels of MRPP2 and MRPP3, the other two subunits of RNase P, were unchanged in fibroblasts from affected individuals (Figure 2A). The loss of MRPP1 protein level correlates with decreased steady-state levels of subunits of complex I (NDUFB8) and complex IV (COXI) (Figure 2B), in agreement with the multiple respiratory chain defects observed in muscle. We used blue native PAGE to determine the effects on the assembly and stability of the respiratory chain complexes^16^ and show a marked decrease of fully assembled complex I and complex IV, with a slight decrease in complex III levels (Figure 2C). The low steady-state levels of mtDNA-encoded proteins was due to impaired mitochondrial protein synthesis in subject fibroblasts as demonstrated by reduced incorporation of ^35^S-labeled methionine and cysteine (Figure 2D).

**Figure 3:**
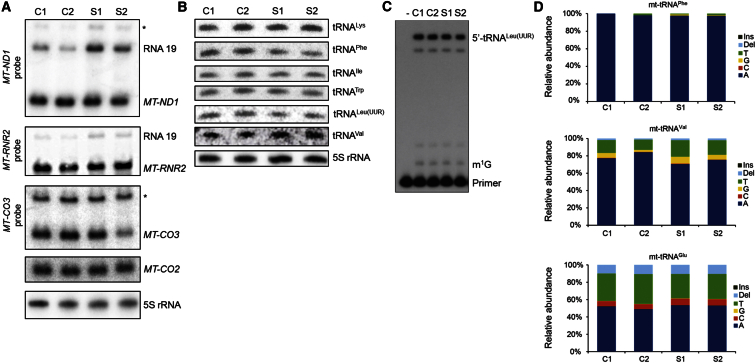
*TRMT10C* Variants Lead to Altered RNA Processing but Do Not Affect Methylation at Position 9 (A) Northern blot analyses of mt-mRNA steady-state levels using radiolabelled probes specific for *MT-ND1*, *MT-RNR2* (16 s rRNA), *MT-CO2*, and *MT-CO3*. Asterisk (^∗^) denotes putative mtRNA precursors. (B) High-resolution Northern blot analyses of mt-tRNAs using radiolabelled oligonucleotide probes against mt-tRNAs with 5S rRNA as a loading control (performed as described previously^33^). (C) Primer extension analysis of m^1^G9 in tRNA^Leu(UUR)^ using a radiolabeled primer (5′- TTATGCGATTACCGGGCTCTGC-3′) annealing 1 base downstream of the modified residue. Primer and 3 μg RNA were denatured at 95°C for 5 min and cooled on ice. Primer extensions were carried out using AMV reverse transcriptase (Thermo Fisher Scientific) at 45°C for 1 hr and stopped by heating at 85°C for 15 min. After ethanol precipitation, the samples were analyzed by fractionation through a 12% polyacrylamide-urea gel and autoradiography. Extension of the primer is partially inhibited by the presence of methylated G9 in mt-tRNA^Leu(UUR)^ leading to the accumulation of a single-base extension product (labeled m^1^G) that is detectable in both control and in case subject RNA at similar levels. (D) Sequencing error rates at position 9 in mt-tRNA^Phe^, mt-tRNA^Val^, and mt-tRNA^Glu^ determined by RNA-seq analysis of mitochondrial RNAs extracted from control and subjects’ fibroblasts. The relative abundance of individual nucleotides and indels generated by the presence of m^1^R9 was analyzed as described previously.^7^

Because MRPP1 is known to be an essential subunit of the mitochondrial RNase P,^6^ which is responsible for 5′ cleavage of mt-tRNAs from the polycistronic mitochondrial transcripts, we investigated whether fibroblasts from affected individuals showed evidence of impaired mitochondrial RNA processing. Northern blot analyses showed an increase in RNA precursor RNA19 when detected with either an *MT-ND1* or *MT-RNR2* probe (Figure 3A). However, the steady-state levels of the mature mRNAs were not significantly affected. No increase in precursors of *MT-CO2* or *MT-CO3* were observed, although the steady-state levels of mature *MT-CO3* appeared to be slightly decreased in subject 2 (Figure 3A).

Processing of mt-tRNAs at the 3′ end is carried out by ELAC2.^7^, ^8^, ^10^ Because both subjects had functional copies of *ELAC2*, it would be expected that the mt-tRNAs would be processed at the 3′ end, but not at the 5′ end, resulting in mt-mRNAs with an uncleaved mt-tRNA at the 3′ end. The resolution of the Northern blots for mt-mRNAs was not sufficient to distinguish between mature mRNA and these pre-processed transcripts, thus, high-resolution Northern blot experiments were performed to assess the levels of mature mt-tRNAs (Figure 3B). Surprisingly, the steady-state levels of mt-tRNAs were not significantly altered in the affected individuals relative to controls, suggesting that the severe mitochondrial translation defect was not due to absence of cleaved mt-tRNAs. However, mt-tRNA^Phe^ and mt-tRNA^Leu(UUR)^ appeared to have slightly lower steady-state levels in subject fibroblasts relative to controls.

To further investigate precursor processing, we carried out RNA-seq analysis of mitochondrial RNA isolated from control and affected individuals. Differential analyses of mt-mRNA and mt-tRNA gene expression in TruSeq library datasets and small RNA library datasets, respectively, revealed no significant differences in mitochondrially encoded mt-mRNA, mt-rRNA, and mt-tRNA levels between the samples (not shown). However, when we investigated the changes in the abundance of reads across the entire mitochondrial transcriptome, we found an increase in the regions that span gene boundaries, where RNA processing is required to release individual mitochondrial RNAs from the precursor transcripts (Figure S1). Together, these data confirm an impairment of mt-tRNA processing efficiency without severe effects on mature mt-mRNA or mt-tRNA steady-state levels. It is possible that the cleavage of mt-tRNAs by mt-RNase P is less efficient in cells harboring *TRMT10C*/MRPP1 variants, but that the mt-tRNAs that are cleaved are very stable, thus retaining steady-state mt-tRNA levels to approximately wild-type levels.

All tRNAs undergo post-translational modification at numerous sites to promote their correct function.^11^, ^17^ The mt-tRNAs are not exceptions and cleavage from the polycistronic mt-RNA transcripts is just one step in their maturation. In addition to their role in RNase P activity, MRPP1 and MRPP2 act as an m^1^R9 methyltransferase.^14^ Methylation of either G or A at position 9 is vital for the correct structure and function of mt-tRNAs.^12^, ^13^, ^18^ Thus, we sought to investigate the impact of the *TRMT10C* variants on m^1^R9 methyltransferase activity in subject fibroblasts. To this aim, we utilized two experimental approaches: (1) primer extension analysis of individual mt-tRNAs during which the reverse transcriptase-mediated extension of a radiolabelled primer is inhibited by the presence of m^1^R9 modification^19^ and (2) RNA-seq analysis, because m^1^ methylation at position 9 has been shown to increase the sequencing error rate at this position.^7^ Primer extension analysis of mt-tRNA^Leu(UUR)^ revealed no difference between control subjects and affected individuals (Figure 3C). Similarly, there was no change in the sequencing error rates between subject and control samples (Figure 3D). These data indicate that the m^1^R9 methyltransferase activity is not affected by the p.Arg181Leu and p.Thr272Ala MRPP1 variants.

**Figure 4:**
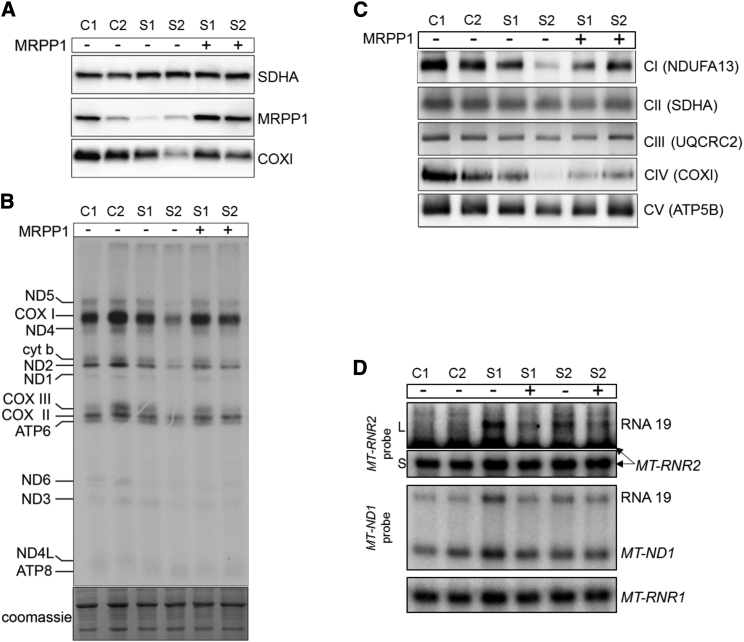
Lentiviral Expression of Wild-Type *TRMT10C* Restores RNA Processing, Expression of mtDNA-Encoded Proteins, and OXPHOS Assembly in Subjects’ Fibroblasts (A) Western blot analysis of fibroblast extracts transduced with lentiviral particles expressing wild-type *TRMT10C*. MRPP1, COXI, and SDHA were detected with specific antisera. SDHA was used as a loading control. (B) In vitro mitochondrial translation in subjects’ fibroblasts transduced with control lentiviral particles or particles expressing wild-type *TRMT10C*. Analyses were performed as in Figure 2C. (C) Blue-native PAGE analysis of OXPHOS assembly in rescued subjects’ fibroblasts performed as in Figure 2B, showing restoration of complex assembly. (D) Northern blot analysis of RNA processing in complemented fibroblasts analyzed as in Figure 3A. For *MT-RNR2* (16S mt-rRNA), short (S) exposure was used with a long (L) exposure shown to highlight the weaker band corresponding to RNA19. Representative images from three independent lentiviral rescue experiments are shown for each panel of this figure.

We have demonstrated that tRNA 5′ processing is affected in fibroblasts from affected individuals with mutant MRPP1, which is consistent with current knowledge of the function of MRPP1 as a component of mt-RNase P. However, to definitively prove that the mitochondrial OXPHOS defect is a consequence of the *TRMT10C* variants, lentiviral rescue experiments were performed to complement the respiratory phenotype expressed in cultured cells. Fibroblast cell lines from affected individuals were transfected with a lentiviral vector carrying a copy of the wild-type *TRMT10C* gene encoding MRPP1. The complemented cell lines displayed increased expression of MRPP1 protein level (Figure 4A), leading to a restoration of mitochondrial translation (Figures 4A and 4B) and normal levels of fully assembled respiratory chain complexes (Figure 4C). Furthermore, the level of mt-RNA precursors, elevated in subject fibroblasts, normalized after lentiviral transduction with wild-type *TRMT10C* (Figure 4D). These data verify the pathogenicity of the c.542G>T (p.Arg181Leu) and c.814A>G (p.Thr272Ala) *TRMT10C* variants, establishing these variants as causative of mitochondrial disease associated with multiple respiratory chain abnormalities.

Prenatal diagnosis has subsequently been offered to both families in subsequent pregnancies after the identification and validation of pathogenic *TRMT10C* variants. In the first family harboring compound heterozygous changes, both *TRMT10C* variants were identified in a later pregnancy after chorionic villus biopsy, leading to termination. In family 2 (homozygous variant), the fetus was heterozygous for the c.542G>T (p.Arg181Leu) *TRMT10C* variant and mitochondrial respiratory chain activities were normal in the chorionic villus biopsy sample (data not shown), supportive of an unaffected clinical status.

Of interest, we noted that both affected individuals showed decreased MRPP1 steady-state protein levels in fibroblasts, although the levels in subject 1 (compound heterozygote variants) had lower levels than subject 2 (homozygous variant), suggesting that the p.Thr272Ala mutant MRPP1 protein is less stable than the p.Arg181Leu mutant. However, despite having higher residual levels of MRPP1, cells from subject 2 exhibited a more severe impairment of mitochondrial protein synthesis resulting in lower steady-state levels of respiratory chain complexes I and IV, implying the p.Arg181Leu mutant protein is more stable but less active than the p.Thr272Ala mutant protein.

The impairment of mt-RNA processing observed in fibroblasts from the affected individuals was not as severe as we anticipated, with steady-state levels of mature mt-mRNAs and mt-tRNAs largely unaffected. However, these data fit well with reports of mutations in other proteins involved with RNA processing, given that mutations in *ELAC2*^20^ and *HSD17B10*^21^ have both been shown to lead to an accumulation of mt-RNA precursors without effects on the levels of mature mt-mRNA and mt-tRNAs. Furthermore, it has been shown that loss of MRPP2 levels leads to a reduction in steady-state levels of MRPP1.^21^, ^22^ Given that the increase in RNA precursors (RNA19) we report was similar to those seen in subjects with deleterious *HSD17B10* (MRPP2) variants (MIM: 300438),^21^ it is possible that decreased MRPP1 protein levels are particularly important for RNA processing because MRPP2 levels were not shown to be diminished in either of the cases documented here (Figure 2B). Conversely, we did not find any evidence of altered m^1^R9 methyltransferase activity in fibroblasts from either affected individual, implying that the observed defect in mitochondrial protein synthesis is due to a decrease in the efficiency of mt-RNA processing rather than any effects on mt-tRNA modification. The m^1^R9 methyltransferase activity is carried out by a stable protein complex of MRPP1 and MRPP2,^14^ whereas the RNA processing requires MRPP3,^6^ which contains the active site of the nuclease activity of RNase P.^23^, ^24^ Therefore, one attractive hypothesis is to suggest that the p.Arg181Leu and p.Thr272Ala TRM10C variants disrupt the interaction between MRPP1 and MRPP3 without affecting the complex with MRPP2, although this requires future investigation.

Knowledge of defects in mt-tRNA processing or modification leading to disease has expanded in recent years including the aforementioned variants in *HSD17B10* (MRPP2)^21^ and *ELAC2*,^20^ as well as mutations in numerous mt-tRNA modifier proteins including *PUS1*^25^ (MIM: 608109), *TRIT1*,^26^ *TRMU*^27^ (MIM: 610230), *TRNT1*^28^ (MIM: 612907), *TRMT5*^19^ (MIM: 611023), *MTO1*^29^, ^30^ (MIM: 614667), and *GTPBP3*^31^ (MIM: 608536). Mutations in *TRMT10C* (MRPP1) can now be added to this growing list as we show that the introduction of wild-type MRPP1 into fibroblasts from affected individuals is sufficient to rescue their mitochondrial defects, confirming these *TRMT10C* variants as pathogenic in mitochondrial disease associated with impaired mitochondrial translation.

## Figures and Tables

**Table 1:** Biochemical and Clinical Findings in Individuals with *TRMT10C* Variants

**ID**	**Sex**	***TRMT10C* Variants**	**OXPHOS Activities in Skeletal Muscle**					**Clinical Features**		
		**cDNA (NM_017819.3); Protein (NP_060289.2)**	**RCC**	**% Mean Enzyme Activity**	**Absolute Values**	**Control Mean (Reference Range)**	**Muscle Biopsy Findings**	**Age at Onset**	**Clinical Course**	**Other Clinical Features and Relevant Family History**
Subject 1^a^	male	c.[542G>T];[814A>G]; p.[Arg181Leu];[Thr272Ala]	I II III IV	48% (↓) 103% 98% 23% (↓)	0.050 0.150 0.543 0.259	0.104 ± 0.036 (n = 25) 0.145 ± 0.047 (n = 25) 0.554 ± 0.345 (n = 25) 1.124 ± 0.511 (n = 25)	COX-deficient, ragged-red fibers	birth	died at 5.5 months	myopathy, hypotonia, sensorineural deafness, liver involvement; elevated serum and CSF lactate levels
Subject 2^b^	female	c.[542G>T];[542G>T]; p.[Arg181Leu];[Arg181Leu]	I II III IV CS	64% (↓) 293% 8% (↓) 6% (↓) 332%	11 88 24 9 319	17 ± 4 (n = 50) 30 ± 7 (n = 50) 303 ± 57 (n = 50) 144 ± 34 (n = 50) 96 ± 18 (n = 50)	ND	birth	died at 5 months	hypotonia, deafness; elevated serum, urine, and CSF lactate levels

a Respiratory chain complex (RCC) activities are expressed as nmols NADH oxidised.min^−1^.unit citrate synthase^−1^for complex I, nmols DCPIP reduced.min^−1^.unit citrate synthase^−1^ for complex II and K.s^−1^.unit citrate synthase^−1^ × 10^3^ for complexes III and IV

b Respiratory chain complex (RCC) activities are expressed as nmol/min/mg protein

## Accession Numbers

The data reported in this paper have been deposited in GEO at GSE79120 and in ClinVar at SCV000264779.0 and SCV000264780.0 for c.542G>T and c.814A>G, respectively.

## Web Resources

ClinVar, https://www.ncbi.nlm.nih.gov/clinvar/
ExAC Browser, http://exac.broadinstitute.org/
GenBank, http://www.ncbi.nlm.nih.gov/genbank/
GEO, http://www.ncbi.nlm.nih.gov/geo/
OMIM, http://www.omim.org/
Phyre2, http://www.sbg.bio.ic.ac.uk/phyre2/html/page.cgi?id=index
PolyPhen-2, http://genetics.bwh.harvard.edu/pph2/
RaptorX, http://raptorx.uchicago.edu
SIFT, http://sift.bii.a-star.edu.sg/

## Supplemental Data

Document S1. Figure S1

Document S2. Article plus Supplemental Data
